# What can a weevil teach a fly, and reciprocally? Interaction of host immune systems with endosymbionts in *Glossina* and *Sitophilus*

**DOI:** 10.1186/s12866-018-1278-5

**Published:** 2018-11-23

**Authors:** Anna Zaidman-Rémy, Aurélien Vigneron, Brian L Weiss, Abdelaziz Heddi

**Affiliations:** 1 Univ Lyon, INSA-Lyon, INRA, BF2I, UMR0203, F-69621 Villeurbanne, France; 2 Department of Epidemiology of Microbial Diseases, Yale School of Public Health, New Haven, CT USA

**Keywords:** Cereal weevil, Tsetse fly, Endosymbiosis, Immunity, Evolution, Insects, Homeostasis

## Abstract

The tsetse fly (*Glossina* genus) is the main vector of African trypanosomes, which are protozoan parasites that cause human and animal African trypanosomiases in Sub-Saharan Africa. In the frame of the IAEA/FAO program ‘*Enhancing Vector Refractoriness to Trypanosome Infection*’, in addition to the tsetse, the cereal weevil *Sitophilus* has been introduced as a comparative system with regards to immune interactions with endosymbionts. The cereal weevil is an agricultural pest that destroys a significant proportion of cereal stocks worldwide. Tsetse flies are associated with three symbiotic bacteria, the multifunctional obligate *Wigglesworthia glossinidia*, the facultative commensal *Sodalis glossinidius* and the parasitic *Wolbachia*. Cereal weevils house an obligatory nutritional symbiosis with the bacterium *Sodalis pierantonius*, and occasionally *Wolbachia*. Studying insect host-symbiont interactions is highly relevant both for understanding the evolution of symbiosis and for envisioning novel pest control strategies. In both insects, the long co-evolution between host and endosymbiont has led to a stringent integration of the host-bacteria partnership. These associations were facilitated by the development of specialized host traits, including symbiont-housing cells called bacteriocytes and specific immune features that enable both tolerance and control of the bacteria. In this review, we compare the tsetse and weevil model systems and compile the latest research findings regarding their biological and ecological similarities, how the immune system controls endosymbiont load and location, and how host-symbiont interactions impact developmental features including cuticle synthesis and immune system maturation. We focus mainly on the interactions between the obligate symbionts and their host’s immune systems, a central theme in both model systems. Finally, we highlight how parallel studies on cereal weevils and tsetse flies led to mutual discoveries and stimulated research on each model, creating a pivotal example of scientific improvement through comparison between relatively distant models.

## Background

Symbiosis is ubiquitous in nature and is a driving force in evolution. Among invertebrates, insects living on nutritionally unbalanced habitats have evolved long-term associations with intracellular mutualistic bacteria (endosymbionts) that complement their diet with metabolic components, including amino acids and vitamins [1–3]. Some well-studied examples in terms of host-symbiont metabolic interactions are the phloem sap feeding-pea aphid *Acyrthosiphon pisum*’s association with the primary endosymbiont *Buchnera aphidicola* [4]; the carpenter ant *Camponotus floridanus*/*Blochmannia floridanus* association [5]; cereal weevils *Sitophilus* sp.’s association with *Sodalis pierantonius* [6]; the mealybug *Planococcus citri*, which associates with *Tremblaya princeps* and *Moranella endobia* [7], the cockroach *Blattella germanica* and its endosymbiont *Blattabacterium cuenoti* [8] and the blood-feeding tsetse fly (Diptera: Glossinidae)’s association with its obligatory, mutualistic symbiont *Wigglesworthia glossinidia* [9].

Insect nutritional endosymbiosis has been largely studied with regards to bacterial genome evolution [10], host ecology [11], and metabolic complementation [12]. In contrast, data on the molecular processes that orchestrate host immune system interactions with symbionts remain scarce [13–18]. Deciphering these relationships is of high interest so as to figure out whether and how host-symbiont coevolution has shaped the host immune response in the context of a chronic bacterial infection [19]. Investigating the mechanisms insect hosts employ to maintain endosymbiont homeostasis will facilitate the identification of specific molecules that disrupt this relationship, thus providing a translational foundation for the development of novel control strategies that target major insect pests and disease vectors.

In the frame of the IAEA/FAO program ‘*Enhancing Vector Refractoriness to Trypanosome Infection*’, which focuses on the tsetse fly (Diptera: Glossinidae) and its interaction with endosymbionts and trypanosomes, the cereal weevil *Sitophilus* (Coleopteran: Dryophthoridae) has been introduced as a comparative system to investigate common and divergent immune regulations and functions involved in symbiont control and distribution. In this review, we highlight common and divergent traits between these two phylogenetically distant insects within the holometabolous group. We discuss how research on the weevil symbiotic association positively impacted studies performed using the tsetse model system, and vice-versa. We focus mainly on the topic of the interactions between the obligate symbionts and their host’s immune system, which is a central theme in both model systems. While cereal weevils house only one endosymbiont, *Sodalis pierantonius* [20–22], tsetse may harbor the commensal bacteria *Sodalis glossinidius* [23, 24] and parasitic *Wolbachia* [25–27], in addition to obligate *Wigglesworthia glossinidia* [9]. The relevance of studying the interaction with the other symbiotic partners in tsetse is evocated in the “perspectives” section.

## Main text

### Quite different yet very much alike

Although dipteran tsetse flies and coleopteran cereal weevils are phylogenetically distant within the holometabolous insects, they exhibit important converging biological features (Table 1). Their prominent common trait is their ability to feed on nutritionally unbalanced media (i.e. vertebrate blood for tsetse and cereal grains for weevils) thanks to their association with endosymbiotic bacteria that complement their diet and fulfill their metabolic needs throughout development. Both the cereal weevil-associated *Sodalis pierantonius* and the tsetse fly-associated *W. glossinidia* belong to the Enterobacteriaceae family of the Gammaproteobacteria class. The obligatory character of these symbionts is attested by: i) ecological features: all natural populations of the two insects house obligate symbionts [6, 20, 21, 28], and ii) functional analyses: experimental depletion of obligate symbionts leads to sterility in tsetse flies [29–31] and to various biological anomalies in cereal weevils [6, 32–35].

**Table 1:** Main characteristics of cereal weevils and tsetse flies and their respective symbionts

Insect host	Cereal weevil	Tsetse fly	
Species	*Sitophilus* spp.	*Glossinidia* spp.	
Applied interest	agronomical pest	Human and livestock disease vector	
Taxonomy	Coleoptera	Diptera	
Reproduction	oviparity	adenotrophic viviparity	
Location of larval development	cereal grain	maternal uterus	
Diet	cereal grains	vertebrate blood	
Symbionts	*Sodalis pierantonius, Wolbachia*	*Wigglesworthia glossinida, Wolbachia, Sodalis glossinidus, gut microbiota, Spiroplasma, Trypanosoma*	
Nutritional endosymbiosis requirement	obligatory in the field	obligatory	
Endosymbionts	*Sodalis pierantonius*	*Wigglesworthia glossinida*	*Sodalis glossinidus*
Phylogeny	Gammaproteobacteria, Enterobacteriaceae	Gammaproteobacteria, Enterobacteriaceae	Gammaproteobacteria, Enterobacteriaceae
Association age	0.03 Myr	50–80 Myr	recent
Metabolic contribution	amino acids - especially Tyr and Phe, vitamins	vitamins	?
Biological Impact	thinner cuticle, decreased fertility, increased developmental rate, loss of flight ability	total loss of fertility	increased longevity, decreased resistance to trypanosomes
Requirement for the host	obligatory in the field	obligatory	facultative
Cultivable	no	no	yes
Genetically manipulable	no	no	yes
Tissue localization	Gut- and ovaries- associated bacteriomes, oocytes	bacteriome and female milk gland	broad tropism except bacteriome
Cellular localization	intracellular (stochastic exception at pupal stage)	mainly intracellular; extracellular in female milk gland	intracellular and extracellular
Transmission	Ovaries (intracellularly)	milk gland (extracellularly)	milk gland and male spermatophore (extracellularly)
Genome size	4.5 Mb	700 KB	4.2 Mb
PG/LPS synthesis pathway	conserved	conserved	conserved (with modification)
Secretion and infection system conservation	type III secretion system	flagellum	type III secretion system

**Fig. 1:**
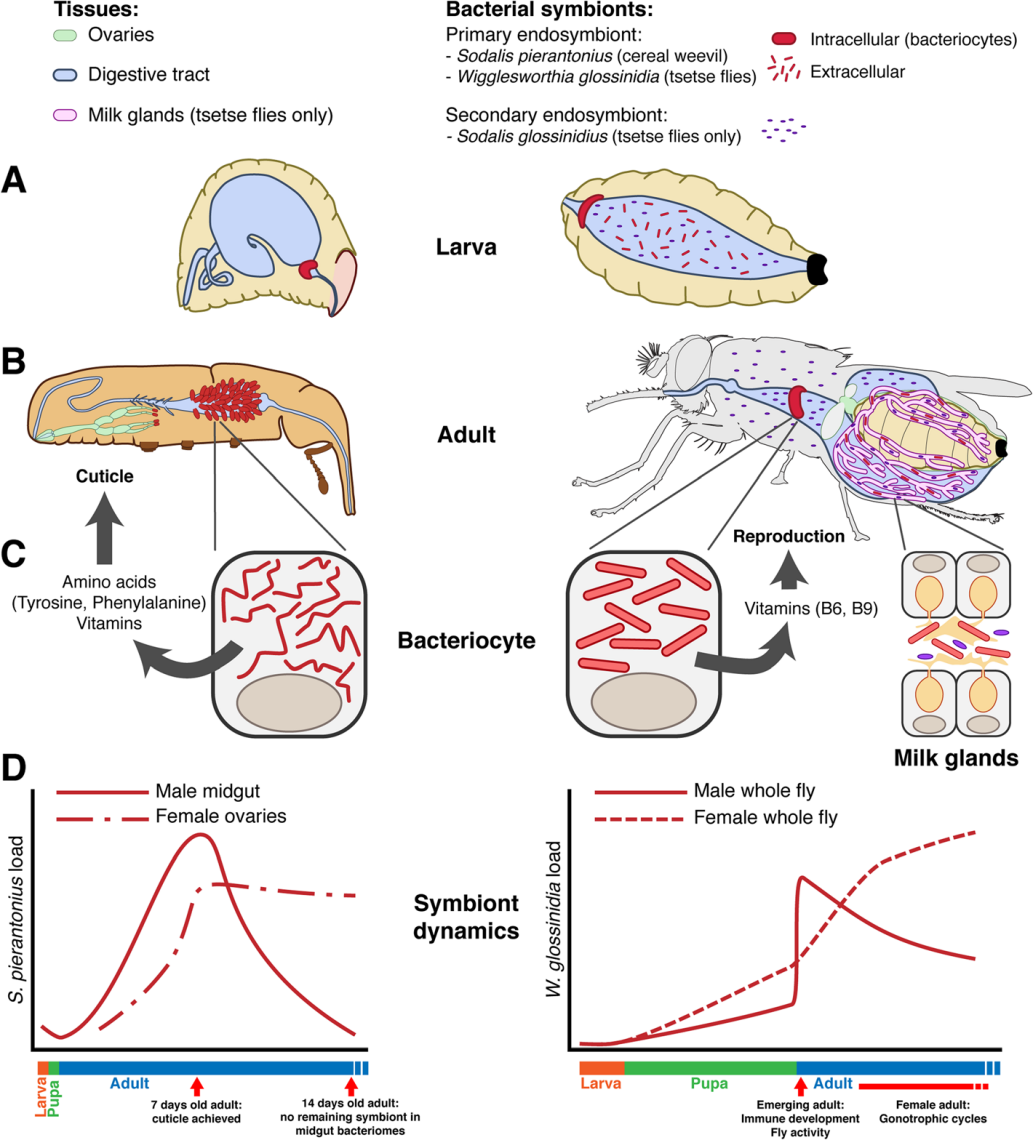
Main endosymbiotic features of cereal weevils and tsetse flies. **a** Schematics of weevil (left) and tsetse (right) larvae. In both models, the obligate symbionts (*Sodalis pierantonius* in the weevil and *Wigglesworthia glossinidia* in tsetse) are present intracellularly in a bacteriome (red) located around the gut (light blue). In tsetse flies, obligate and facultative symbionts (*Sodalis glossinidius*) can be found extracellularly in the midgut lumen, both originating from maternal milk secretions provided as nourishment to developing intrauterine larvae. **b** Schematics of weevil (left) and tsetse (right) adults. In both models, the obligate symbionts are present intracellularly in bacteriomes (red). In the weevil, bacteriomes are present at the apex of midgut mesenteric caeca (light blue), as well as at the apex of female ovaries (light green), from which maternal transmission occurs. In tsetse flies, the obligate symbiont is also located intracellularly in a bacteriome (red) located around the midgut (blue) as well as extracellularly in the lumen of the milk glands (pink). In tsetse, the facultative symbiont is distributed intra- and extracellularly throughout the whole fly, including the lumen of the milk glands. Both obligate and facultative symbionts are maternally transmitted through milk feeding. **c** Schematics of weevil (left) and tsetse (right) bacteriocytes. *S. pierantonius* is an elongated bacterium that exhibit high size variability. *W. glossinidia* is a large rod-shaped bacterium. In both model, the obligate symbionts located in midgut bacteriocytes supports their host with nutrients that are used to build exoskeleton and for reproduction in the weevil and tsetse, respectively. **d** Obligate symbiont growth dynamics in the weevil (left, adapted with permission from [35]) and in tsetse (right, adapted with permission from [100]). In both models, bacterial load dynamics follow the main biological needs of the host. In the weevil, the obligate endosymbiont’s density increases considerably during exoskeleton synthesis. After cuticle completion, the endosymbionts located in mesenteric caeca are eliminated and recycled. Female weevils keep a stable load of endosymbiont in their ovaries for transmission. In adult male tsetse, *W. glossinidia* density increases dramatically immediately following eclosion (likely in response to the onset of mating activities), and then slowly declines thereafter. In adult females, *W. glossinidia* density constitutively increases, likely as a reflection of the metabolically costly process of nourishing intrauterine larvae

This deep integration with their insect host’s physiology is also remarkable at the cellular level, as both obligate symbionts are housed within specialized host cells named bacteriocytes, which group into an organ called the bacteriome [6, 36] (Fig. 1). Interestingly, the strict intracellular status of these symbionts was recently challenged in both insects with the recurrent presence of extracellular *W. glossinidia* in the milk gland of female tsetse [37, 38] and sporadic externalization of *S. pierantonius* at a time-restricted step during metamorphosis [39], during which time the unique larval bacteriome is transitioning into multiple adult bacteriomes [35, 40].

*W. glossinidia* supplements its host’s diet mainly by producing essential vitamins that are absent from vertebrate blood [41–43]. This function infers that, during their 50–80 million years of coevolution [44], tsetse has developed a metabolic dependence upon *W. glossinidia* [43]. Likewise, *S. pierantonius* provides its weevil host with vitamins and cofactors. However, this bacterium appears especially important as a source of amino acids, in particular tyrosine and phenylalanine [35, 45–49]. These distinct, symbiont-specific metabolic contributions, which are clearly deduced from their respective genome sequences [22, 50], are likely related to the unique biological traits of their insect hosts. Specifically, tsetse flies employ a reproductive strategy called ‘adenotrophic viviparity’. During this process the female retains one offspring within her uterus throughout its entire larval development, and provides it with nourishment in the form of glandular milk secretions [51] (Fig. 1). Female tsetse require *W. glossinidia*-derived vitamins and co-factors to sustain the energetically costly process of milk production and thus reproductive fitness [42]. The cereal weevil, on the other hand, presents an oviparous mode of reproduction. Females lay eggs inside cereal grains, and both larval and pupal stages occur within the grains. While protected during these stages, the survival of emerging adults relies in large part on the thickness of their cuticle, which determines their ability to avoid desiccation and overcome pathogen challenges while outside of the grains. The building of a strong cuticle, a characteristic feature of coleopterans, requires a huge amount of tyrosine and phenylalanine amino acids, which are the precursors of the 3,4-dihydroxyphénylalanine (DOPA) amino acid involved in the cuticle melanization and sclerotization (see below). Endosymbiont contribution to host cuticle synthesis through aromatic amino acid supply appears to be a convergent strategy among beetles [52–54].

In addition to metabolic studies, the importance of symbionts on the host physiology has been assessed in both models by generating insects artificially deprived of symbionts (aposymbiotic insects), either through antibiotic treatments (tsetse; [29, 31]) or a defined heat/humidity treatment (weevil; [55]). Aposymbiotic tsetse flies are reproductively sterile and impaired in their ability to resist infection with trypanosomes as well as normally non-pathogenic *E. coli K12*. [29–31, 56, 57]. Conversely, aposymbiotic cereal weevils remain fertile enough to allow for the maintenance of aposymbiotic strains under lab conditions. However, aposymbiotic cereal weevils exhibit a thinner cuticle, are less fertile, develop more slowly, and lose their ability to fly [6, 34, 35]. These phenotypes demonstrate the importance for both insects to maintain their association with mutualistic symbionts.

Weevils harbor only one obligate endosymbiont, *S*. *pierantonius*. However, tsetse flies can also house the facultative symbiont *Sodalis glossinidius* (Fig. 1)*.* The functional contribution of this bacterium, which is present in all colonized flies and some wild populations [24, 58–60] has not been definitely determined to date. Analysis of *S. glossinidius’* genome showed an overlap of metabolic pathways with those encoded by *W. glossinidia’s* genome [50, 61]. Despite this apparent redundancy, depletion of *S. glossinidius* decreases tsetse longevity [62], suggesting that this bacterium plays a role in mediating its host’s overall fitness. Contrary to *W. glossinidia* and *S. pierantonius*, *S. glossinidius* can be grown in culture media [63, 64]. This may reflect the fact that *S. glossinidius* is constitutively found both intracellularly and extracellularly in tsetse, indicative of the fact that it has conserved the capacity to survive outside the eukaryotic cell, similarly to its free-living bacterial relatives. Noteworthy is that despite being part of the same genus, *S. pierantonius* and *S. glossinida* have evolved distinct ecological adaptations, which begs the question of what differs between their genomes that allows these different adaptations.

Although each insect has its own ecological and biological specificities, the tsetse fly and the cereal weevil present important similarities with regard to their relationships with endosymbiotic bacteria. Most importantly, like many other insects, both rely on their obligate symbiont to facilitate their development and survival. This raises several questions related to how these insects have evolved to integrate and transmit their microbial partners across host generations, and to the immune strategies that regulate the symbiont load and spatial distribution during the insect’s life cycles.

### Immunity and symbiosis

During the last decade, insect immune interactions with endosymbionts has been thoroughly deciphered in several other insect systems [15], e.g. the pea aphid [65, 66], the carpenter ant [67–69] and, the leafhopper [70]. Tsetse flies and cereal weevils exhibit two common features that have likely impacted the evolutionary shaping of their immune system: i) they both have co-evolved with endosymbiotic bacteria for thousands of host generations, hence their immune systems must be able to accommodate the chronic presence of dense symbiont populations, and, ii) both insects must be able to trigger an effective immune response following infection by pathogenic microbes. This is particularly true at the imago stage as both tsetse and weevil’s larval stages are protected from pathogens, either “hidden” inside the uterus of the female tsetse fly, or inside a sterile grain endosperm in the case of the cereal weevil. These constraints on an immune system that must retain the ability to both “tolerate” beneficial microbes and strike against pathogens, or symbionts that would escape bacteriocytes, is a shared problem among insects that host endosymbiotic bacteria. At least three main, non-exclusive ‘strategies’ have evolved to address these issues in relevant insect host-symbiont model systems.

#### Strategy 1 - *The evolution of symbiont antigens that would normally be recognized by host immune receptors*

Because of their strictly vertical transmission and the lack of genetic recombination with free-living bacteria, insect endosymbionts present a typical coevolution with their host, which often results in massive pseudogenization of the bacterial genome followed by a loss of genes and their subsequent functional pathways [10, 22, 71]. Such pseudogenized genes often include those involved in host cell infection processes, including secretion systems, and/or synthesis of cell wall components, including lipopolysaccharides (LPS) and peptidoglycans (PGN), that can elicit an immune response in many insects [72]. Remarkably, comparative genomics showed that none of the weevil and the tsetse symbionts have lost the genes required for LPS and PGN synthesis [22, 41], although *S. glossinidus*’ genome encodes a truncated LPS that does not include an O-antigen [73]. It would be of interest to confirm this genomic data with biochemical characterization of the symbiont cell wall.

#### Strategy 2 - *The loss of host immune mechanisms that could trigger a response to and damage the symbiont*

One example of such a strategy is provided by the pea aphid *Acyrthosiphon pisum*. The genome of this insect host lacks many elements commonly found in the canonical IMD pathway, including the Peptidoglycan Recognition Protein (PGRP) immune receptor family and downstream antimicrobial peptides (AMPs; [65, 74]). Transcriptomic studies indicate that IMD pathway-associated gene erosion may occur in other insects, including leafhoppers [70]. In cereal weevils and tsetse flies, genomics and/or transcriptomics data reveal well-conserved IMD-like pathways similar to those described in *Drosophila* and *Tribolium* [75, 76]. Furthermore, both insects mount robust immune responses upon infection with free-living bacteria [77–82] and trypanosomes in the case of tsetse [78, 79]. Tsetse and weevil immune responses following intrathoracic challenge with *E. coli* (in the tsetse [18]) or its PG derivative (TCT; in the weevil [83]) are *pgrp-lc*/*relish*-dependent [18] and *imd*/*relish*-dependent [83], respectively. In weevils, *S. pierantonius* injection into the hemolymph also results in the induction of a cocktail of AMP encoding genes [80]. However, under standard conditions, when the endosymbiont is present in bacteriocytes only, the host systemic immune response remains basal. These data indicate that endosymbionts are recognized as microbe intruders when present into the hemolymph, and attests that endosymbionts are tolerated in the bacteriocyte cells only. While similar experiments have to our knowledge not been conducted (or not published) in the tsetse fly with *W. glossinidia*, the injection of *S. glossinidus* into tsetse hemocoel also triggers a potent immune response. *S. glossinidus* seems to have evolved other means of escaping the immune system effectors, since it was found to resist antimicrobial peptides Attacin [77] and Diptericin [84], and PGRP-LB bactericidal action [18]. Some of these mechanisms rely on the bacteria outer membrane protein A (OmpA), which for instance allows *S. glossinidus* to form biofilms in the tsetse gut [85].

#### Strategy 3 - *Symbiont compartmentalization*

In addition to germ cells, bacterial partners are often restricted to bacteriocyte cells, themselves often grouped into a bacteriome organ. This seclusion from the rest of the host’s tissues occurs in both weevils and tsetse flies, and would notably protect symbionts from the host systemic immune system while promoting the control of symbiont population density [86]. Symbiont compartmentalization also prevents costly permanent immune activation in response to the bacterial partner, which could be detrimental for the host fitness. The tsetse and weevil endosymbionts discussed in this review have retained some genes that are known in other systems to be required for host cell infection, which is the first step of the compartmentalization process. These genes include those that encode type III secretion systems in *S. pierantonius* and *S. glossinidius* [87, 88], and the flagellum apparatus in *W. glossinidia* [41]. The conservation of these elements in weevil and tsetse symbionts could be due to: i- *S. pierantonius* has established its endosymbiosis with the cereal weevil only recently (less than 0.03 MY; [89]), following the replacement of the weevil endosymbiont ancestor *Candidatus* Nardonella [90–92], and its genome is actively decaying, as attested by high pseudogenization rate; ii- both weevil and tsetse symbionts are found extracellularly, which leads to the presumption these bacteria are capable of reinfecting host cells.

Based on these observations, researchers working on weevil and tsetse models are similarly interested in better understanding 1) the cellular and molecular mechanisms that facilitate obligate symbiont seclusion within host bacteriocytes, 2) the molecular mechanisms that prevent the activation of the immune responses by bacterial partners, and 3) whether and how endosymbiotic evolutionary constraints have shaped immune genes and pathways for symbiont maintenance and control.

Below we further discuss how exchanging scientific information about both models has contributed significantly to stimulating new discoveries relative to these current research questions.

### Molecular basis of obligate symbiont compartmentalization

One essential feature of the host-symbiont relationship is the control of the bacteria with respect to both their location and density within the host. As discussed above, symbiont compartmentalization can be considered as a ‘biological strategy’ that allows host organisms to manage beneficial symbionts within a limited space. This compartmentalization protects the symbionts from direct exposure to the host’s systemic immune response and is a reflection of functional adaptation found in several insect-symbiont systems, including the two we focus on in this review. Stringent symbiont control within the niche relies on distinct molecular mechanisms tailored to each insect host.

Molecular mechanisms that underlie weevil control of *S. pierantonius* were initially characterized using the suppressive subtractive hybridization (SSH) technique on larval bacteriomes [80, 93], followed more recently by RT-qPCR and RNA-sequencing (RNA-seq) at different developmental stages [82, 94]. The larval stage was studied first, as it represents a ‘steady state’ in terms of endosymbiosis homeostasis in that the endosymbiont population increases slowly and no bacteriocyte cell modification is observed by cell imaging. These approaches revealed several genes putatively involved in bacteriome physiology and immunology. The weevil bacteriome mounts a limited immune response, with few immune effectors being expressed under normal physiological conditions, i.e. presence of the endosymbionts and absence of infection with exogenous bacteria [80, 93]. Remarkably, this organ expresses only one AMP coding gene, *coleoptericin A* (*colA*) [80, 82]. The remaining AMP coding genes are expressed at almost undetectable levels, or not at all, which presumably facilitates endosymbiont survival within the bacteriome. This genetic expression program is designated the ‘bacteriome internal response’, i.e. the transcriptional reaction of the bacteriome in response to the presence of the endosymbiotic [86].

Biochemical studies, in vitro assays and in vivo functional analyses demonstrated that *colA* expression is essential for endosymbiont control. In vitro incubation of *E. coli* with weevil ColA peptide impairs bacterial cell division and leads to cell gigantism [95]. While *S. pierantonius* population is pleomorphic and includes long and filamentous cells under standard conditions, inhibition of *colA* gene expression by RNA interference (RNAi) induces a significant size reduction of the bacterial cells. Importantly, RNAi-induced reduction of *colA* expression resulted in a loss of spatial control of endosymbionts. Under these conditions, the bacteria were able to exit the bacteriocytes and to invade surrounding tissues [83, 95]. These results, along with the relatively high amount of ColA peptide observed at the bacteriome border, led to the conclusion that ColA acts as a molecular ‘guard’ that prevents endosymbionts from leaving the bacteriome, thus ensuring homeostasis of the symbiotic system [83, 95]. Moreover, ColA specifically targets weevil endosymbiont cytokinesis but does not inhibit DNA replication. This mechanism leads to the production of giant polyploid bacterial cells and is hypothesized to be the result of the protein’s specific interaction with the bacterial chaperonin GroEL [95, 96]. Endosymbiont polyploidy could be advantageous for the insect host’s physiology because it may result in an increased abundance of bacterial transcripts that encode beneficial metabolites.

Hence, endosymbiont regulation by ColA is likely an adaptive feature that arose as the result of host–endosymbiont coevolution. More specifically, ColA spatially restricts endosymbionts within bacteriocytes and inhibits their cytokinesis without impairing the bacterial metabolic activity nor ability to supply the host with nutritional components. This adaptation highlights the concept of ‘endosymbiont domestication’ [96]. Interestingly, *colA* basal expression in the bacteriome is under control of the same IMD-like pathway that regulates AMP expression upon bacterial infection [83]. In line with this, RNAi-driven *relish* knock-down sufficiently recapitulates the loss-of-control phenotype observed with *colA* RNAi [83]. This suggests that the same pathway can be involved in immune responses leading both to pathogen elimination and fine-tuned control of endosymbiotic partners.

In the tsetse model, recent systematic RNA-seq and metabolomics-based analyses of the bacteriome have provided valuable insight into the organ’s function [43, 97]. In contrast to weevils, these analyses were performed on adult females, and resulted in the identification of several molecules involved in the bacteriocyte physiology, including sodium/potassium pumps, a metalloprotease and proteins related to vesicular transport/exocytosis. In terms of immunity, only two characterized immune-related genes were identified, a lectin and PGRP-LB [43]. Tsetse PGRP-LB possesses antibacterial activity against *E. coli* [98], and this activity could be also effective against *W. glossinidia* and thus participates in the control of the symbiont. As Wang et al. (2012) clearly demonstrated that PGRP-LB is secreted by the bacteriome and the milk gland, one remaining question is whether its antibacterial activity could act on the symbiont present inside this organ (for example if some proteins remained inside the bacteriocytes), or mainly function on symbionts present extracellularly in the milk gland lumen [98]. Moreover, noteworthy is the fact that no AMP encoding transcript(s) was identified in these data. However, this outcome may be biased by the size cut-off chosen for the transcript analysis, and therefore further studies are required to determine the level of AMP expression in tsetse’s bacteriome. More generally, it would be relevant to determine whether AMP encoding genes are expressed by tsetse’s bacteriome following a systemic or oral infection of the fly, in order to assess the organ’s immunogenic potential. Interestingly, although no ColA orthologs is encoded in tsetse’s genome [99], *W. glossinida* is also polyploid and contains from 3 to 23 genome copies [100], suggesting that control of this symbiont may also rely on the inhibition of bacterial cytokinesis.

### Modulation of symbiont load according to the host physiological needs throughout life

In both cereal weevils and tsetse flies, host-symbiont interactions vary across the host life cycle in response to its physiological needs (Fig. 1). How the host controls and adjusts the symbiont load according to these physiological needs at different developmental stages is a central question in this research field.

At metamorphosis, the weevil larval bacteriome dissociates and multiple small bacteriomes are formed at the apex of the adult mesenteric caeca [35, 40]. During the first week of adult life, these mesenteric bacteriomes grow drastically in size and the endosymbiont population quickly expands more than 10-fold [35]. Strikingly, bacteriomes then regress rapidly during the second week of adulthood until the endosymbionts are completely eliminated. Adults live up to 6 months in the laboratory under these conditions. In females the ovarian-associated symbiont population is protected from this elimination process. The rapid increase in symbiont density in the first days of adulthood parallels the host’s need for the amino acids tyrosine and phenylalanine, which are produced by the endosymbiont and transformed by the insect into 3, 4-dihydroxyphenylalanine (DOPA), a compound required for strengthening and stabilizing the newly synthesized cuticle [35]. Once the formation of the new adult cuticle is achieved, endosymbionts are rapidly eliminated.

C*olA* expression in gut bacteriomes of young adults is correlated with symbiont density, and the bacteria remain intracellular during the whole elimination process, thereby avoiding tissue inflammation and systemic immune activation [94]. These findings suggest that, similarly to the larval stage, ColA keeps targeting and regulating endosymbiont cell division as long as bacteria are present in adults. Remarkably, expression of the other AMP coding genes remains basal and is not correlated with bacterial replication dynamics [94]. This, in addition to the high transcript abundance of the negative immune regulator *pirk* during the first weeks of adulthood, suggests an active clamping of the local immune system that may be relevant not only for endosymbiont tolerance, but also for permitting their rapid growth at the initial phase of the adult stage [94]. Therefore, from larval development to adulthood, endosymbiont compartmentalization allows the host to control its bacterial partner to accommodate its metabolic needs.

Taking into consideration the low expression of AMP encoding genes during the endosymbiont elimination phase, AMPs are unlikely to be involved in this dynamic process. Symbiont elimination instead occurs via apoptosis and autophagy, the latter of which is a conserved cellular mechanism allowing eukaryotic cells to recycle cell components and organelles and to preserve cellular homeostasis [35, 94]. These cellular processes may allow the host to minimize the cost inherent to symbiont growth in the initial adult phase. The autophagy-dependent recycling of endosymbiont cell components would enable the host to recover a part of the energy invested in endosymbiont growth, control and maintenance. Remaining questions to address include: i/ what are the mechanisms that underlie drastic symbiont growth at the beginning of adult stage; ii/ the synchronization of endosymbiont growth and clearance with the host physiology; and, iii/ whether the endosymbiotic modulation is modified under environmental or biological (e.g. infections) stresses.

Tsetse fly development from egg to adult stage takes approximately 40 days, and adults can live up 3 months. Interestingly, symbiont density is also dynamic throughout the fly’s life cycle, which indicates that adjustment of symbiont density to their host’s physiological needs likely occurs in this model system as well. *W. glossinida* and *S. glossinidus* density is steady during larval development. At metamorphosis, however, both *W. glossinida* and *S. glossinidus* populations increase, and the increase becomes dramatic in the first days of adult life [100]. Two weeks later, *S. glossinidius* density decreases dramatically in males and females, as does *W. glossinidia* density in adult males. However, these decreases do not result in a complete elimination of the bacteria, as seen in the weevil [35]. In female tsetse, *W. glossinidia* increases less rapidly at the beginning of adulthood, but thereafter progressively increases during the first 4 weeks of adult life [100]. *W. glossinidia* is essential for tsetse fertility [31]. The temporal and tsetse sex-specific dynamics of this symbiont’s replication program in the fly likely reflect the metabolically burdensome requirements associated with nurturing intrauterine larvae [42]. Female tsetse generate eggs and nourish larvae throughout adulthood and thus require constant metabolic input from *W. glossinidia*. Moreover, females transmit the symbiont to their progeny through milk gland secretions [37, 38], and as such, maintenance of this endosymbiosis is required for transmission to subsequent generations.

In this context, it would be interesting to precisely analyze the tissue-specific dynamics of symbiont density in female tsetse. The cellular and molecular mechanisms that modulate symbiont growth dynamics are to date not well described. However, the correlation between the expression profile of PGRP-LB and *W. glossinidia*’s density, combined with the in vitro activity of the protein against Gram-negative bacteria, suggests that PGRP-LB could be involved in this process [18, 98]. Comparing the molecular profile in a tissue-specific and sex-specific manner could provide additional clues as to the molecular players involved. For instance, this information could tell us whether modulation of the gut bacteriome population is sex-dependent, and if so, whether the female milk gland presents an immunocompetent phenotype that accounts for sex-specific differences. Several non-exclusive hypotheses related to regulation of symbiont density could be advanced, including involvement of sex hormones, the initiation of blood feeding at adulthood and/or differences in abiotic/environmental conditions (e.g., humidity, temperature, etc.) present between intrauterine larval, pupal (underneath leaf litter or soil) and adult environments (deposited pupae).

### Avoiding the triggering of a detrimental immune response due to the symbiont presence

Symbiont compartmentalization within specialized cells is an evolutionary adaptation that likely protects the bacteria from detrimental host immune responses. In line with this, the weevil’s potent systemic immune response against exogenous Gram-negative bacteria does not impact endosymbiont density within the bacteriome [81]. Similarly, induction of tsetse’s immune responses following challenge with exogenous bacteria or parasitic trypanosomes also does not impact symbiont load [100]. Nevertheless, two questions remain to be addressed.

#### Question 1 - *How is bacteriocyte gene expression modulated to allow for the maintenance of endosymbiont load?*

Despite the presence of massive amounts of bacteria inside bacteriocytes, all AMP encoding genes, with the exception of *colA*, are expressed at low levels in this tissue [80, 82]. This restrained local immune response in bacteriocytes reflects host ‘tolerance’ towards the bacterial partner and is likely essential for endosymbiont maintenance. This response also raises the question of whether conventional canonical immune pathways are functional in this organ. This question was addressed by challenging weevil larvae with exogenous free-living bacteria and subsequently monitoring the expression of immune encoding genes [81]. This treatment resulted in the upregulation of a cocktail of AMP encoding genes, indicating that the bacteriome is reactive to exogenous bacterial infections [81]. In contrast to the ‘internal’ immune response that is directed towards endosymbionts, this bacteriocyte response was qualified as ‘external’, as exogenous bacteria from the outside of the bacteriome trigger the local response. Remarkably, this external response does not seem to interfere with the endosymbiont load [81].

Thus, the bacteriome presents an immune program adapted to maintaining endosymbiotic homeostasis under standard conditions while retaining the ability to mount an immune response against exogenous microbial intruders. The exact mechanisms limiting the expression of AMP encoding genes other than *colA* in the bacteriome in the presence of *S. pierantonius* exclusively (and absence of exogenous infections) are still unknown, especially considering that all AMPs are *imd*/*relish*-dependent in this tissue [83]. This finely tuned, selective response may involve specific modulation of promoters that regulate expression of AMP encoding genes, or epigenetic modifications such as DNA or histone methylation. Similarly, molecular mechanisms that regulate bacteriome immune responses in tsetse remain largely unexplored.

#### Question 2 - *Does seclusion of endosymbionts within bacteriocytes prevent their detection by systemic host immune receptors?*

In other words, is the compartmentalization strategy sufficient to explain the absence of host systemic immune responses against endosymbionts, which could negatively impact the host either directly through collateral damage caused by immune effectors or indirectly via costly metabolic expenditure? This question has been partially addressed in both weevils and tsetse flies by investigating *pgrp*-*lb* encoding genes.

PGRP-LB is a negative regulator of the immune response in *Drosophila melanogaster* [101]. By cleaving PG, *Drosophila* PGRP-LB down-modulates the IMD pathway in the absence of an exogenous infection (gut commensals only) and after systemic and oral infection [101]. Interestingly, *pgrp-lb* orthologues are highly expressed in the bacteriome of both cereal weevils [39] and tsetse flies [18]. The expression of these genes correlates with the extracellular status of symbionts: *pgrp-lb* expression increases at weevil pupal stage; its tsetse orthologue is not only expressed in the bacteriome but also in the milk gland of female tsetse flies [39, 98].

PGRP-LB’s role in *Drosophila* led to the hypothesis that the protein would similarly act as a negative regulator that limits weevil and tsetse symbiont detection by cleaving their PG. This hypothesis was further substantiated by the fact that weevil and tsetse’ PGRP-LB both contain the key amino acids required for the catalytic activity in orthologous proteins [18, 93]. It was proposed that PGRP-LB would degrade the PG from *S. pierantonius* and *W. glossinidia* symbionts, avoiding its putative recognition upstream of an Imd-like pathway. This relies on the hypotheses that in tsetse and the weevil, like in the fruit fly, PG potently elicits the immune response through its recognition by the PGRP-LC receptor. This in turn activates the Imd pathway leading to the production of antimicrobial peptides, a process that is downregulated by PGRP-LB through its enzymatic activity of PG degradation. Evidence toward this model is the phenotype observed after RNAi-driven functional analysis of tsetse PGRP: suppression of *pgrp-lb* expression induced *attacin A* (whole fly analysis; [18]). Correspondingly, this treatment also leads to a decrease in *W. glossinidia* density, suggesting that PGRP-LB prevents host immune responses that are detrimental to *W. glossinidia* under homeostatic conditions. This result suggests that in the absence of PGRP-LB, either the bacteriome itself expresses a detrimental antimicrobial response, or the systemic immune response somehow impacts symbionts located in the bacteriome. Alternatively, the inhibitory effect observed on *W. glossinidia* density may reflect an antimicrobial response triggered in the milk gland. Indeed, PGRP-LB is secreted by tsetse’s milk gland, and *attacin A* expression increases in this niche in parallel with decreased local *W. glossinidia* density [98].

Other questions still await clarification on the function of the PGRP-LB in the weevil and tsetse fly systems. For example, it remains to be determined whether bacteriome-derived PGRP-LB only functions locally in this tissue, or if it acts systemically to regulate extra-bacteriome immune responses. Additionally, it would be relevant to elucidate the precise biochemical activity of the protein on different PG substrates, as well as the molecular basis of the protein’s intriguing trypanocidal activity [98]. Finally, addressing whether the weevil orthologue exhibits a similar function would provide valuable insight into the evolutionary mechanisms of immune regulation across distant insect orders (coleopterans and dipterans), and in endosymbiotic (tsetse, weevil) versus non-endosymbiotic (Drosophila) insects.

### Does endosymbiosis shape the host immune system?

The impact of endosymbiosis on immune system maturation is well described in the tsetse fly model system and has recently been addressed in the cereal weevil. Weiss et al. (2011) demonstrated that young adult tsetse flies (3 days post-eclosion) perish following systemic challenge with 1000 CFU of *E. coli* while mature adults (8 days post-eclosion) survive [102]. The authors went on to demonstrate that *W. glossinidia* played an important role in determining these differential infection outcomes, as mature adults that underwent their entire developmental program in the absence of this bacterium (these flies are designated ‘aposymbiotic’) were as susceptible to *E. coli* infection as were young wild-type individuals. Interestingly, aposymbiotic tsetse are also unusually susceptible to infection with pathogenic African trypanosomes [31, 57]. Importantly, *W. glossinidia*’s influence on tsetse’s immune system appears to be developmental, as flies that harbor the bacterium throughout all immature stages, but are then deprived of it during early adulthood, still present a functional immune system thereafter [102].

Aposymbiotic tsetse are significantly impaired in their ability to mount a typical systemic humoral immune response, as reflected in the lower expression of AMP genes after *E. coli* challenge. These flies also present a highly dysfunctional cellular immune system and thus lack circulating and sessile hemocytes. These blood cells are responsible for phagocytizing foreign intruders and participating in the fly’s melanization response [56, 102]. The absence of hemocytes in aposymbiotic tsetse likely reflects their significantly decreased expression of *serpent* and *lozenge* during larvigenesis [56, 102]. These transcription factors regulate early stages of dipteran hematopoiesis, or blood cell differentiation [103]. Further analyses revealed that differentiation of one tsetse hemocyte subtype, crystal cells, which are involved specifically in the dipteran melanization cascade [104], is regulated by *W. glossinidia*-induced odorant binding protein 6 (OBP6) [105]. The gene that encodes this protein is significantly up-regulated in the gut of early instar tsetse larvae upon ingestion of the obligate symbiont. OBP6 production is necessary and sufficient to sequentially induce expression of *lozenge*, which actuates crystal cell formation [105, 106]. Moreover, the authors observed a similar effect of *Drosophila’s* larval gut microbiota, strongly suggesting a conservation of this hematopoietic regulatory mechanism among these two dipteran species [105]. Future studies in tsetse flies will certainly aim to address the precise mechanisms of *W. glossinidia* activity on *obp6* expression, including whether it relies on a direct, active signal from the symbiont, or an indirect effect, possibly through symbiont input towards tsetse host metabolism.

In contrast to the tsetse model system, endosymbiosis appears to have less of an effect on cellular immune system development in *S. oryzae* [107]. This weevil houses five larval hemocyte morphotypes, and their abundance and morphometry drastically change along insect development, with a significant increase in hemocyte number and size occurring between the 3rd larval instar and early pupae. Hemocytes participate to the weevil cellular immune response, as these cells respond to challenge with pathogenic exogenous *E. coli*. However, in contrast with the tsetse system, symbiotic-dependent modulation of the global weevil hemocyte population was not observed, as wild-type and aposymbiotic larvae and young pupae housed similar hemocyte numbers [107].

The different mechanisms that underlie cellular immune system development in these two model systems likely evolved to reflect their distinct ecologies. First of all, the cereal weevil and tsetse fly do not face the same evolutionary constraints in terms of immunity as they harbor very different symbionts. Cereal weevils interact exclusively with *S. pierantonius* and *Wolbachia*, while tsetse flies associate with *W. glossinidia*, *S. glossinidius*, a taxonomically diverse population of environmentally acquired enteric bacteria and African trypanosomes [9, 28]. Noteworthy is the observation that although cereal weevils exhibit melanization at cuticular wound sites, crystal cells have yet to be detected in this insect (unpublished data). Weevil (or more generally Coleoptera) and tsetse fly (or more generally Diptera) hemocytes may not assume the same functions in terms of melanization, especially because these insects may not be facing similar challenges that require melanization as a counteractive immune response. For instance, evidence suggests that tsetse flies could have been the target of pupal parasitism [108, 109], while cereal weevils are presumably protected from such parasitism by the grain they inhabit and then by their strong adult cuticle. A second point that could explain the distinct mechanisms regulating cellular immunity development in the two models is the fact that *S. pierantonius* and *W. glossinidia* are two distinct bacterial species, which may have evolved different abilities to manipulate their host organisms. In this regard, it is interesting to note that *S. glossinidus*, a closer relative of *S. pierantonius* when compared to *W. glossinida*, is not able to stimulate tsetse immune system development [56, 105]. Nevertheless, more thorough analyses on the association between symbiosis and hemocyte functions (e.g. phagocytic efficiency after infection by exogenous microbes, the transcriptomic or proteomic profiles of these immune cells) are required to fully determine if weevil endosymbionts impact the development and function of their host’s cellular immune system. Additionally, characterization of endosymbiont impact on weevil systemic humoral immunity should also be examined.

### Perspectives: Other players in the game

In this review, we have voluntary eluded a conspicuous difference presented by the two models, which is the number of their reported symbiotic interactions. The cereal weevil harbors one unique mutualistic endosymbiont, *S. pierantonius*, and in some cases, *Wolbachia* as well [6]. However, *Wolbachia*-free weevil lines are always used when performing functional studies, and we thus do not know if or how this bacterium impacts weevils’ interactions with the obligate endosymbiont *S. pierantonius*. Intriguingly, to date no gut microbiota has been identified in the weevil, despite an extensive search using sequencing and microscopy (Heddi’s lab unpuplished data). In contrast, the tsetse fly houses several partners other than obligate *W. glossinidia*, including *S. glossinidius* [64], *Wolbachia* (Proteobacteria: Rickettsiacecae) [110, 111], *Trypanosoma* parasites and a taxonomically variable and complex environmentally acquired enteric microbiota [58, 59, 85, 102, 112–114]. Similarly, some tsetse species are also associated with *Spiroplasma* [115], suggesting other interactors less numerous in density remain to be identified. Studying the potentially complex network of interactions between the tsetse hosts and its symbionts is relevant for at least three reasons: i) the co-existence of these interacting partners has likely influenced the evolution of tsetse fly immune system. One example could be the acquisition of antiparasitic function by the PGRP-LB molecule [98]; ii) tsetse symbionts could contribute to the extended phenotype of the fly in terms of defense mechanisms [116], including against the Trypanosoma, or to its susceptibility suggested by correlations observed in the field [114, 117], but not yet confirmed by experimental approaches in the lab; iii) *S. glossinidus*, *Wolbachia* and the enteric microbiota represent potentially fruitful tools to manipulate the tsetse fly and its ability to spread Trypanosoma [27, 118–120]. Indeed, in an effort to develop novel cost-effective strategies to impair the spread of trypanosomiasis, it was suggested that symbionts, especially *S. glossinidius*, could be exploited using paratransgenesis to reduce trypanosome cycle success and transmission through the tsetse fly [121]. This method could be used to complement the established Sterile Insect Technique (SIT) approach [122].

## Conclusion

Along this review, we have highlighted a number of research foci for which the results obtained in one model has enriched the general understanding of endosymbiosis and immunity interactions, and stimulated further discovery in the other model. In some cases, mechanistic similarities are evident between how the two insects manage their symbiotic partners. Conversely, while physiological distinctions do exist, research performed using these two models has generated experimental hypotheses that have fostered fruitful research on the other. Behind the important techniques and methodological exchanges between the two research communities, it appears that interconnected science on the two models can stimulate the discoveries made on both, as the two models “respond” to each other through a similar questioning. More than explicit answers to these questions, they give their respective research communities hints of interesting paths to follow, even if these paths may lead to different places.

## Abbreviations

AMP: Antimicrobial peptide
Col: Coleoptericin
DOPA: 3,4-dihydroxyphenylalanine
*E. coli*: *Escherichia coli*
Obp6: Odorant binding protein six
PGRP: Peptidoglycan recognition protein
RNA-seq: RNA-sequencing
*S. glossinidus*: *Sodalis glossinidus*
*S. pierantonius*: *Sodalis pierantonius*
TCT: Tracheal cytotoxin
*W. glossinida*: *Wigglesworthia glossinida*
